# The Effect of Solution Casting Temperature and Ultrasound Treatment on PEBAX MH-1657/ZIF-8 Mixed Matrix Membranes Morphology and Performance

**DOI:** 10.3390/membranes12060584

**Published:** 2022-05-31

**Authors:** Elsa Lasseuguette, Louise Fielder-Dunton, Qian Jian, Maria-Chiara Ferrari

**Affiliations:** 1 School of Engineering, University of Edinburgh, Robert Stevenson Road, Edinburgh EH9 3FB, UK; e.lasseuguette@ed.ac.uk (E.L.); s1725507@sms.ed.ac.uk (L.F.-D.); 2 EaSTCHEM School of Chemistry, University of St Andrews, St Andrews KY16 9ST, UK; qj4@st-andrews.ac.uk

**Keywords:** mixed matrix membranes, PEBAX, ZIF-8, CO_2_ permeability, crystallinity, casting temperature

## Abstract

Approximately two-thirds of anthropogenic emissions causing global warming are from carbon dioxide. Carbon capture is essential, with membranes proving to be a low cost and energy-efficient solution to alternative technologies. In particular, mixed matrix membranes (MMMs) can have higher permeability and selectivity than pure polymer membranes. The fabrication conditions affect the formation of defects within the membranes. In this work, MMMs were created using a PEBAX MH-1657 polymer and a ZIF-8 filler. The effect of casting plate temperature, varying from −5 °C to 50 °C, and the effect of ultrasound treatment time (80–400 min) and method (filler solution only, filler and polymer combined solution only and filler solution followed by combined solution) were investigated, aiming to reduce defect formations hence improving the performance of the MMMs. SEM images and permeation tests using pure CO_2_ and N_2_ gas, replicating flue gas for carbon capture, were used to investigate and compare the membranes morphology and performance. The results indicated that the MMMs maintained their permeabilities and selectivities at all tested casting temperatures. However, the neat PEBAX membranes demonstrated increased phase separation of the polyamide and polyether oxide phases at higher temperatures, causing a reduction in permeability due to the higher crystallinity degree, confirmed by DSC experiment. The MMMs fabricated at low ultrasound times displayed a large amount of aggregation with large particle size causing channeling. At high ultrasound times, a well-dispersed filler with small filler diameters was observed, providing a high membrane performance. Overall, defect-free membranes were successfully fabricated, leading to improved performance, with the best membrane resulting from the longest ultrasound time reaching the Robeson bound upper limits.

## 1. Introduction

Membrane technology demonstrates considerable potential in the field of gas separation due to its low energy requirements, excellent mechanical properties, ease of fabrication and scale-up [1,2,3]. However, polymer membranes have a trade-off between selectivity and permeability, and have an empirical upper limit known as the Robeson bound [4]. To overcome this limitation, mixed matrix membranes (MMMs), which combine a polymer with a filler, create an opportunity to surpass the Robeson bound. By using two materials with different transport properties, such membranes have the potential to combine synergistically the easy processability of polymers and the superior gas-separation performance of filler materials, improving the selectivity and permeability. The addition of the filler alters the packing structure and free volume of the polymer, thus changing the performance of the membrane.

However, the transport properties through the MMMs are strongly dependent on the nanoscale morphology of the membranes and the polymer/particle interface [5]. For instance, poor compatibility between filler and polymer results in voids or caving at the interface. These defects provide a low resistance and a non-selective route for the gas to bypass the filler, resulting in higher CO_2_ and N_2_ gas permeability but in lower selectivity. Another possible source of defects at the interface is fillers aggregation or the formation of cluster, which either prevent, reduce or slow the gas passing through the membrane. Aggregation results in reduced permeability and, in some cases, reduced selectivity [6].

In terms of the matrix, many kinds of polymer materials have been used to fabricate MMMs, such as polyimides [7,8,9], polyacetylenes [10,11], polymers with intrinsic microporosity [12,13,14], polysulfones [15], or copolymers such as PEBAX [5,16]. PEBAX, also known as poly(amide-b-ethylene oxide), is a thermoplastic block copolymer created from alternating polyether oxide (PEO) and polyamide (PA) blocks. The PEO block (rubbery phase) behaves as an amorphous permeable region owing to its high chain flexibility, whereas the PA block (glassy phase) behaves as a rigid, dense phase [17]. The crystalline PA provides mechanical strength and thermal stability due to physical crosslinks enhancing chain mobility [18,19]. PEBAX is an excellent polymer for CO_2_/N_2_ separations for carbon capture due to the interactions between CO_2_ and the polar oxygen in the PEO. These interactions result in high selectivity (40 to 50 [19]) for CO_2_ over non-polar N_2_ gas. Moreover, the high chain flexibility and the presence of polar and nonpolar groups allows better compatibility with inorganic fillers [7]. For the fillers, MOFs are considered excellent fillers thanks to their high porosity and internal surface area [20,21]. Among these, recently ZIF-8, a zeolite imidazolate framework, gained attention as nanoscale fillers for MMMs because of their molecular sieving effect, facile synthesis, and good compatibility with polymers [22,23]. The ZIF-8 filler, also known as Basolite Z1200 or zeolitic imidazolate framework-8, consists of a zinc cation surrounded by four imidazolate rings forming a sodalite structure [24] inducing large cavities and small apertures (11.6 Å and 3.4 Å diameter, respectively). ZIF-8 has a pore size of 0.34 nm, which is between the kinetic diameter of CO_2_ (0.33 nm) and N_2_ (0.36 nm). As a result, ZIF-8 acts as a molecular sieve, further emphasizing its CO_2_ selective nature [23].

Fabrication conditions (such as drying time, solvent evaporation temperature, and ultrasound treatment) largely affect the nanoscale morphology of the MMMs, and it is crucial to optimize them to create a defect-free membrane to achieve their best performance. Many studies have noted the importance of casting temperature on membrane morphology and performance [25,26,27]. For instance, Karamouz et al. [26] showed that a higher evaporation rate resulted in a more dispersed phase of the membrane, which led to more permeable and selective membranes. On the other hand, a fast evaporation time can also decrease the permeability since the time is too short for the polymer chains to rearrange, leading to chain entanglement and potentially causing barriers within the membrane’s pores [27]. Wahab et al. [28] investigated the effect of casting temperature on CO_2_/N_2_ gas separation using a PEBAX MH-1657 polymer and PVDF thin layer composite membrane. They found that the CO_2_ gas permeability decreased with the increasing casting temperature above 40 °C due to a decrease in FFV from polymer chain rearrangements.

In order to obtain a homogenous MMM, ultrasound treatments have to be used in MMM formation [29]. Increasing ultrasound time results in decrease of the filler’s aggregation [30] and size [31,32], which minimizes defect formation. Ahmad et al. [33] studied the impact of this treatment on the separation performances of MMM based on Polysulfone mixed with 15% wt of 4A zeolite. Increasing the ultrasound time resulted in a decrease in CO_2_ permeability due to the better dispersion of the filler giving a more homogeneous membrane, thereby decreasing aggregation and reducing channeling of gases through the membrane. This reduction also decreased N_2_ permeability causing an increase of CO_2_/N_2_ selectivity with ultrasound time.

The purpose of our study was to investigate the effect of casting plate temperature and ultrasound treatment time to minimize the formation of defects, thereby improving the MMMs separation performance. The MMMs were created using a PEBAX MH-1657 polymer and a ZIF-8 filler at 10% wt loading. The ZIF8 content was chosen according to the literature [34,35,36]. By comparing MMMs and neat polymer membranes fabricated, differences in permeability and selectivity were evaluated using the Robeson bound plot. The permeability of the membranes was measured using a constant volume, varying pressure permeation experiment, and the morphology and crystallinity of the membranes were assessed using SEM and AFM images, and DSC experiments, respectively.

## 2. Materials and Methods

### 2.1. Materials

**Figure 1:**
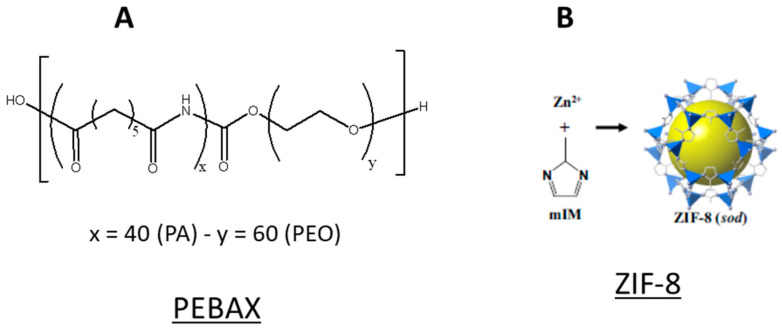
Chemical structure of PEBAX MH1657 (**A**) and ZIF-8 (**B**) [24].

The copolymer PEBAX-MH-1657 in elliptic pellets was supplied by Arkema (named PEBAX in the manuscript and reported in Figure 1). The copolymer was comprised of 60 wt.% polyether oxide (PEO) and 40 wt.% polyamide-6 (PA-6). ZIF-8 with an average particle size of 4.9 μm and was purchased from Sigma Aldrich. Absolute ethanol (Fisher Scientific, Loughborough, UK) was used as the solvent along with deionized water. The nitrogen and carbon dioxide gas used for the permeation experiments (purity 98%) were procured from BOC. All materials were used as received, without further modification or purification.

### 2.2. Membrane Preparation

PEBAX MH1657 (0.75 g) was dissolved in water/ethanol mixture (3.5 g/7.9 g) at 80 °C under reflux for 3 h. In the meantime, ZIF crystals [0.08 g (10%)] were suspended in a 70/30 wt.% water/ethanol mixture by ultra-sonication. Then, the two solutions were combined and sonicated for 1 h before casting. The resulting solution was poured onto a casting plate and cast by doctor blade with a gap of 70 μm. Then, the membrane was covered with a top-drilled box and let dry for 36 h at ambient temperature.

**Figure 2:**
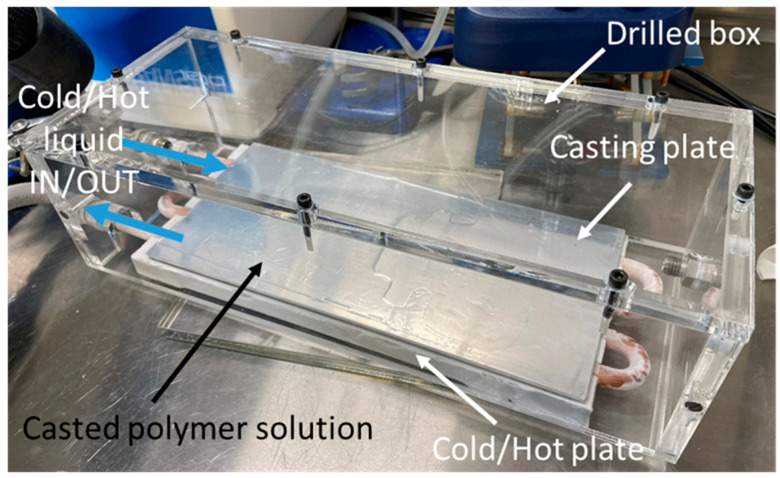
Membrane preparation.

For reviewing the effect of casting temperature, the temperature of the casting plate was heated/cooled to the following temperatures: −5, 10, 25, 35 and 50 °C using a varying temperature hot/cold plate (Figure 2) connected to a temperature regulator bath (MAGIO MS-1000F, Julabo, Stamford, UK). 

For reviewing the effect of the ultrasound treatment, the time and method used were varied. The following treatments were performed: four-hour ultrasound treatment (UT) on the combined solution (4hC), four-hour UT of the filler followed by briefly stirring for one minute with the polymer solution (4hF), one-hour UT on the filler solution and 20 min combined UT (1hF 20mC), three hours UT on the filler solution followed by one-hour UT on the combined solution (3hF 1hC), and finally five hours UT for the filler followed by one hour and forty minutes UT on the combined solution (5hF 1h40C). The temperature of the ultrasound bath was maintained at 50 °C.

### 2.3. Characterization

#### 2.3.1. SEM (Scanning Electron Microscope)

The membranes were examined with a JSM-IT100 (JEOL, Tokyo, Japan) operating at 10 kV. Before SEM analysis, the samples were fractured in liquid nitrogen and then sputtered with a layer of 12 nm gold to form a conductive surface.

#### 2.3.2. XRD Experiment

Films. The crystalline structure of fabricated MMMs were determined by measurements in flat plate geometry, which were conducted on a PANalytical Empyrean diffractometer (Malvern Panalytical Ltd., Malvern, UK) with Cu X-ray tube (Cu Kα1) and X’celerator RTMS detector with a detection 2θ range of 3° to 40°.

Powder. The crystalline structure of ZIF-8 was determined by Powder X-ray diffraction (PXRD) at Stoe STAD I/P diffractometer (Mo Kα1 X-radiation, λ = 0.70930 Å) with a detection range of 1.3 to 18.7°, and could be transferred into Cu X-radiation by applying equation:

$$\sin^{-1}\left(\frac{\sin\left(\frac{2\theta_{\mathrm{Mo}}}{2}\right)}{0.460416}\right)\times 2=2\theta_{\mathrm{Cu}}$$

#### 2.3.3. DSC Experiment

Differential Scanning Calorimetry (DSC) experiments were carried out using a Mettler Toledo TGA/DSC3^+^ (Zurich, Switzerland). Small pieces of membranes (approx. 20 mg) placed in 70 μL ceramic pans were heated under an air flow (40 mL min^−1^) from 25 to 250 °C at a heating rate of 10 °C min^−1^.

#### 2.3.4. AFM (Atomic Force Microscopy)

Atomic force microscopy (AFM) measurements were taken on the membrane surface using a JPK NanoWizard 4XP mounter on a Zeiss Axio Observer microscope (Oberkochen, Germany). The height and slope of the images were obtained in tapping mode under ambient conditions. A Bruker TESPG-V2 probe (Ettlingen, Germany) was used with a nominal spring constant of 42 Nm^−1^ and a nominal fundamental resonance frequency of 320 kHz.

#### 2.3.5. Gas Permeation

**Figure 3:**
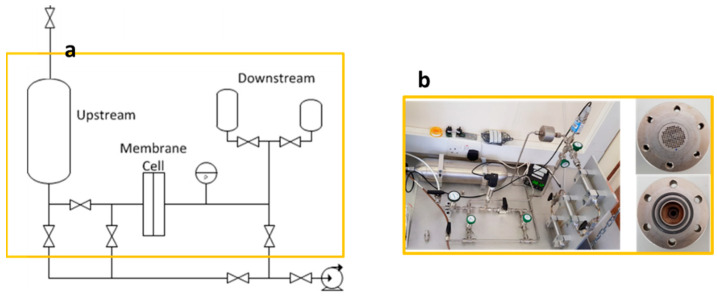
Permeation rig: (**a**) schematic; (**b**) rig with the cell.

Gas permeation tests using nitrogen and carbon dioxide were conducted using a variable pressure/constant volume permeation setup, as shown in Figure 3. Details on the permeation test can be found in a previous publication [13,14].

Permeability was obtained from the evolution of pressure on the downstream side. The permeability coefficient, *P*, was determined from the slope of the pressure vs. time curve under steady state condition using the following Equation (1):

(1) $$P=\frac{l}{A}\frac{V_{down}\;}{P_{up}RT}\left[{\left(\frac{dP_{down}}{dt}\right)}_{ss}\right]$$

where *l* is the membrane thickness, *A* is the membrane area, *V_down_* is the downstream volume, *P_up_* is the upstream pressure, *P_down_* is the downstream pressure, *T* is the temperature recorded during analysis and *R* is the gas constant.

The time lag, *θ*, which is the time required for the gas penetrants to diffuse through the membrane was used to determine the diffusivity coefficient *D* (Equation (2)).

(2) $$D=\frac{l^{2}}{6\theta }$$

The solubility coefficient, *S*, for the gas in the polymer was evaluated indirectly, assuming the validity of the diffusion-solution mechanism (Equation (3)):

(3) $$S=\frac{P}{D}$$

The ideal selectivity between two gas species *i* and *j* is the ratio of the two single gas permeabilities (Equation (4)).

(4) $$\alpha_{ij}=\frac{P\left(i\right)}{P\left(j\right)}$$

## 3. Results

### 3.1. Effect of Casting Temperature

#### 3.1.1. Morphology of Neat PEBAX

**Figure 4:**
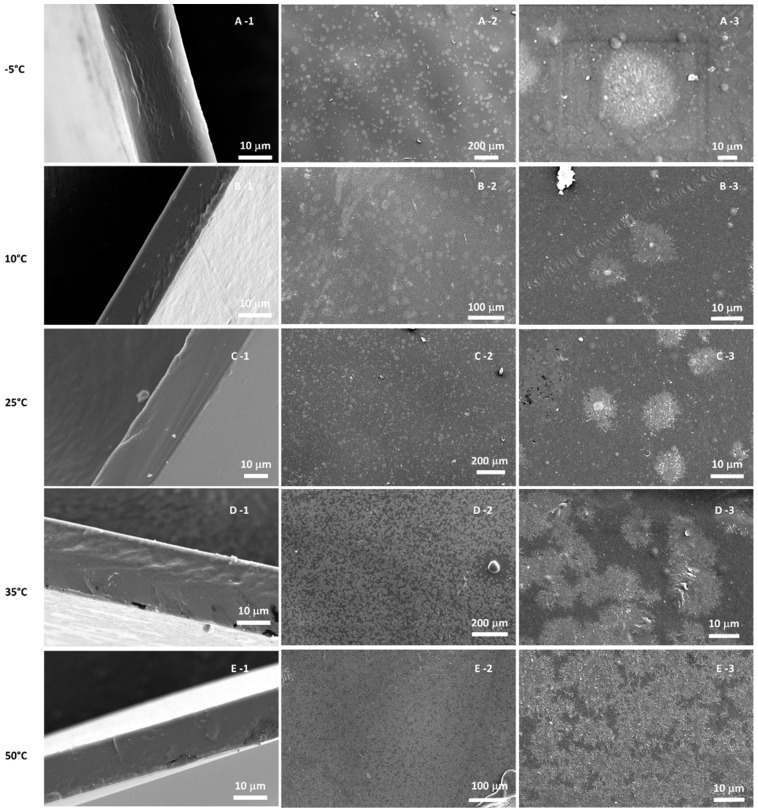
SEM cross sectional (-1) and surface (-2, -3) images from PEBAX cast at different temperatures (A: −5 °C, B: 10 °C, C: 25 °C, D: 35 °C, E: 50 °C).

SEM images of the prepared neat PEBAX membranes cast at different temperatures are presented in Figure 4.

The cross-section morphology displayed a homogeneous and dense structure across the membranes, without differences depending on the casting temperature. On contrary, the surface of PEBAX was impacted by the casting temperature. Spherulite formations could be observed, and their size and number were different according to the temperature. Phase separation occurred between the two phases of the PEBAX polymer membrane (PEO and PA). The glassy PA phase crystallized out from the rubbery PEO phase and created the appearance of an ‘ice crystal’ formation on the surface on the membrane. Under a greater magnification of the spherulite, long and slender lamellae crystals could be observed as displayed in Supplementary Materials Figure S1.

**Figure 5:**
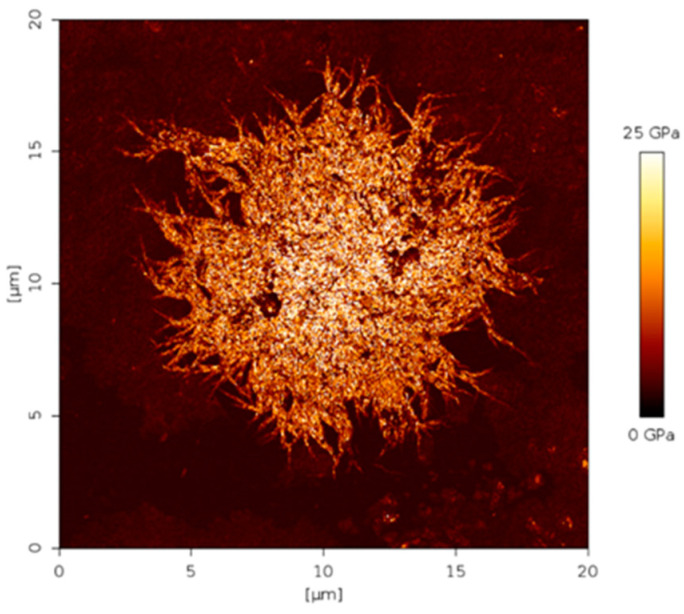
Young’s modulus AFM image of spherulite crystal in PEBAX membrane.

Confirmation of the phase separation of PEBAX to form the PA spherulites was gained using AFM imaging. Figure 5 shows a spherulite formed on the PEBAX membrane cast at room temperature. It displays Young’s modulus, which defines the different viscoelastic properties between the PA and PEO phases. The PA phase was glassy (hard); therefore appearing brighter in the image, whereas PEO was rubbery (soft) and appears darker. This result is also confirmed for PEBAX in literature by Rahman et al. [37]. The image indicates the spherulite to be brighter, with the lamellae crystals visible within the structure, confirming that the spherulite is formed from the PA phase.

The spherulite crystals formed at the different casting temperatures varied in size and shape. As shown in Figure 4, as the casting temperature increased, the spherulites were more numerous and covered almost the whole surface of the membrane. Actually, at higher temperature, the growth rate was higher [38,39]. The thickness of the lamellae also increased with temperature.

The XRD pattern of neat PEBAX (Figure S2) displayed peaks at 2θ values of 21° and 23.8° attributed to the crystalline PA phase [40,41]. With the increase in casting temperature, the peaks appeared more intense. This suggests an increase in crystallinity with the temperature, which corroborates with the SEM images.

#### 3.1.2. Morphology of MMMs

**Figure 6:**
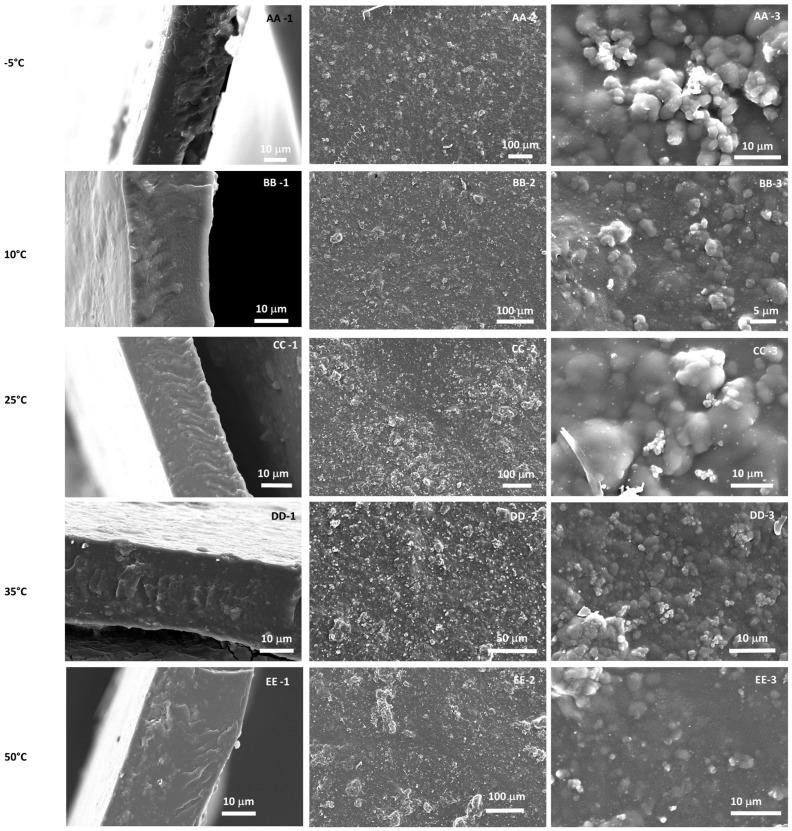
SEM cross sectional (-1) and surface (-2, -3) images from PEBAX-ZIF-8 cast at different temperatures (AA: −5 °C, BB: 10 °C, CC: 25 °C, DD: 35 °C, EE: 50 °C).

SEM images of the cross section and surface of MMMs cast at different temperatures are presented in Figure 6.

The surface appeared rough with the filler exposed at the surface, appearing white on the membrane. The appearance of white filler at the top of the membrane indicated small amounts of irreversible aggregation of the ZIF-8 filler. The same white pattern has been observed in other studies [40]. No visible PA phase separation was noticed on the MMMs. This can be explained by a disruption of the polymer chains with the addition of ZIF-8, which modifies the crystallinity of the polymer [41] and hinders the PA crystallization. Zheng et al. [34] also observed the absence of spherulites with more than 10%wt ZIF-8 in PEBAX.

XRD patterns of the MMMs (Figure S2) indicate a lower degree of crystallinity for the polymeric matrix part, with PEBAX peaks less intense than in the neat one. On the contrary, the crystallinity structure of ZIF-8 was well retained, and all the characteristic peaks for ZIF-8 were still present in the MMM.

The cross-section morphology displayed a dense, homogeneous membrane with good filler dispersion. No obvious macrovoids were observed between PEBAX and ZIF-8, indicating a great compatibility between the filler and the matrix, which is necessary for having high separation performances.

#### 3.1.3. Crystallinity

Thermal analysis of the membranes provided information about the modifications induced to the matrix by the casting temperature and the addition of the filler. From the DSC experiments, it was possible to evaluate the degree of crystallinity of the PA phases in PEBAX. The thermograms of neat PEBAX and MMM at different casting temperatures are reported in Figure S3, with the melting peak corresponding to the PA segments around 207 °C, similar to those in the literature [27,41]. From this melting point, it is possible to calculate the degree of crystallinity of the PA phase ($X_{c}$) by using the following Equation (5):

(5) $$X_{c}=\frac{\Delta H_{f}}{\Delta H_{f}^{*}}\times 100$$

where Δ*H_f_* (J/g) is the enthalpy of fusion corresponding to the area of the melting point and ΔH*_f_ is the enthalpy of fusion when the polymer phase is purely crystalline (23 J/g for PA [41]). 

**Figure 7:**
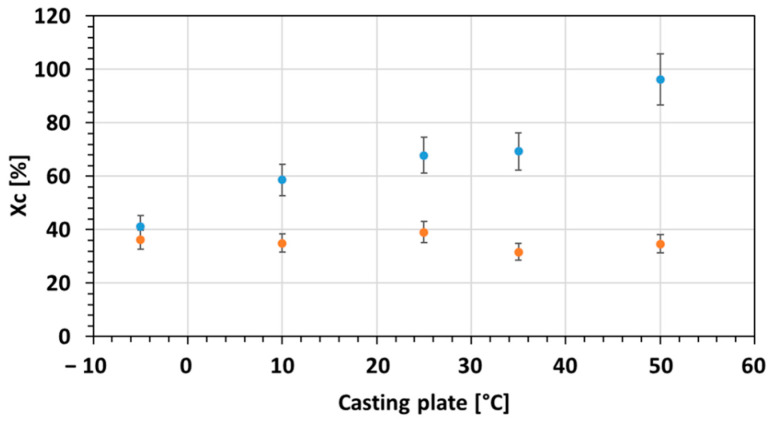
Crystallinity degree of neat PEBAX (Blue) and MMMs (Orange) according to the casting plate temperature (PA phase).

The crystallinity degrees are shown in the Figure 7.

With the increase of the casting plate temperature, the degree of crystallinity for the PA phase within neat PEBAX increased, as the SEM images and the XRD patterns suggested.

Concerning the MMM, a decrease in $X_{c}$ was observed when ZIF-8 was added to the matrix. Actually, the addition of ZIF-8 disrupts the polymer chain arrangement inducing a decrease in crystallinity. Meskhat et al. [41] noticed the same phenomenon with PEBAX mixed with ZIF-8 and ZIF-67. Contrary to the neat PEBAX, there was no variation of the $X_{c}$ with the casting temperature. The same result was noticed with the XRD patterns. As shown in Figure S2, the XRD patterns of MMM were similar with increasing casting temperature.

#### 3.1.4. Separation Performances

##### Permeability and Selectivity

**Figure 8:**
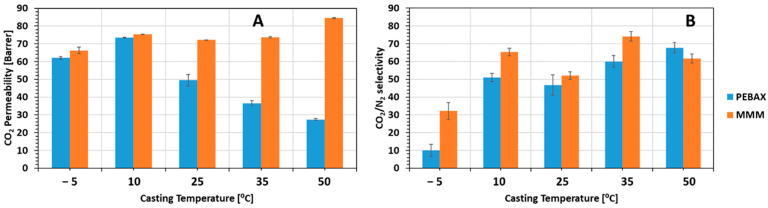
Variation of CO_2_ permeability (**A**) and CO_2_/N_2_ selectivity (**B**) as a function of the casting temperature for neat PEBAX (Blue) and MMMs (Orange).

Figure 8 illustrates the results for the permeability and selectivity for the neat PEBAX and MMMs cast at different temperatures.

For neat PEBAX cast at ambient temperature, the CO_2_ permeability and CO_2_/N_2_ selectivity were similar to the literature data [5,16,18], 50 Barrer and 47, respectively. By increasing the temperature of the casting plate, we noticed a decrease in CO_2_ permeability and an increase on CO_2_/N_2_ selectivity, y 46% and 68%, respectively, up to 50 °C. This behavior is induced by the increase of the crystallinity degree of PEBAX. As explained before, the increase of the casting plate temperature resulted in the formation of PA crystalline spherulites. The reduction of permeability was larger in Nitrogen due to its larger kinetic diameter compared to CO_2_.

By decreasing the temperature of the casting plate, we noticed the opposite with an increase of CO_2_ permeability and a decrease of the CO_2_/N_2_ selectivity. At −5 °C, the polymer solution freezes in a molten state, without any order, which induces larger gas pathways, higher permeability and lower selectivity.

The MMMs created succeeded in improving both permeability and selectivity compared to the neat PEBAX membranes for all the temperatures. Adding ZIF-8 into the PEBAX increased the number of voids between the polymers chains, increasing the FFV compared to the neat PEBAX. The higher FFV is due to the dispersed ZIF-8 within the PEBAX disrupting the polymer chain packing and linking, resulting in a higher permeability. The high CO_2_ selectivity was due to the ZIF-8 selective adsorption of CO_2_ compared to N_2_, increasing the CO_2_ solubility compared to the neat PEBAX membrane [41]. 

As shown in Figure 8, the casting plate temperature did not have an important impact on the CO_2_ permeability of MMMs due to the fact the ZIF-8 modified the crystallinity of the matrix and prevented the formation of spherulites. 

##### Diffusivity and Solubility

**Figure 9:**
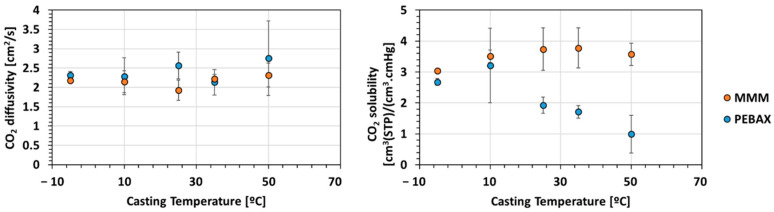
Variation of CO_2_ diffusivity and solubility as a function of the casting temperature for PEBAX (Blue) and MMMs (Orange).

CO_2_ solubility and diffusivity coefficients were determined. Table S1 summarizes permeability, diffusivity and solubility coefficients. Diffusivity coefficients were calculated from Equation (3), while solubility coefficients were obtained from Equation (4). Figure 9 shows the variation of the diffusivity and solubility with the casting plate temperature for PEBAX and MMMs.

The addition of MOF induced a slight decrease of diffusivity coefficient and an increase of the solubility coefficients for CO_2_. The solubility enhancement is explained by a higher CO_2_ solubility value for ZIF-8 due to its higher number of accessible adsorption sites for CO_2_ compared to neat PEBAX [41]. This improvement contributed to the increase of the CO_2_ permeability.

The casting plate temperature had no impact on the CO_2_ diffusivity, as it was stable (in range of the measurement error). On the contrary, the variation of the temperature affected the CO_2_ solubility with two different behaviors for neat PEBAX and MMMs. Increasing the temperature of the casting plate induced an increase of the CO_2_ solubility for the MMM and a decrease for the neat PEBAX. For semi-crystalline polymers, the solubility can be described by the following Equation (6) [42]:

(6) $$S=\left(\frac{100-X_{C}}{100}\right)S_{a}$$

where $X_{c}$ is the degree of crystallinity and *S_a_* is the solubility for a purely amorphous polymer.

Thus, the solubility tends to decrease with increasing the crystallinity degree. As showed before, the increase on the casting plate temperature induced a higher crystallinity degree of PEBAX and, by consequence, a decrease in solubility.

For the MMM, the addition of ZIF prevented the crystallization of PA and induced a decrease of the crystallinity degree. However, the increase in casting plate temperature had no impact on the CO_2_ solubility for the MMM.

These results show that for neat PEBAX, increasing the casting plate temperature induces an increase CO_2_/N_2_ selectivity but a decrease of CO_2_ permeability, whereas for the MMM the casting temperature has no impact on the separation performance.

### 3.2. Effect of Ultrasound Treatement

#### 3.2.1. Morphology

**Figure 10:**
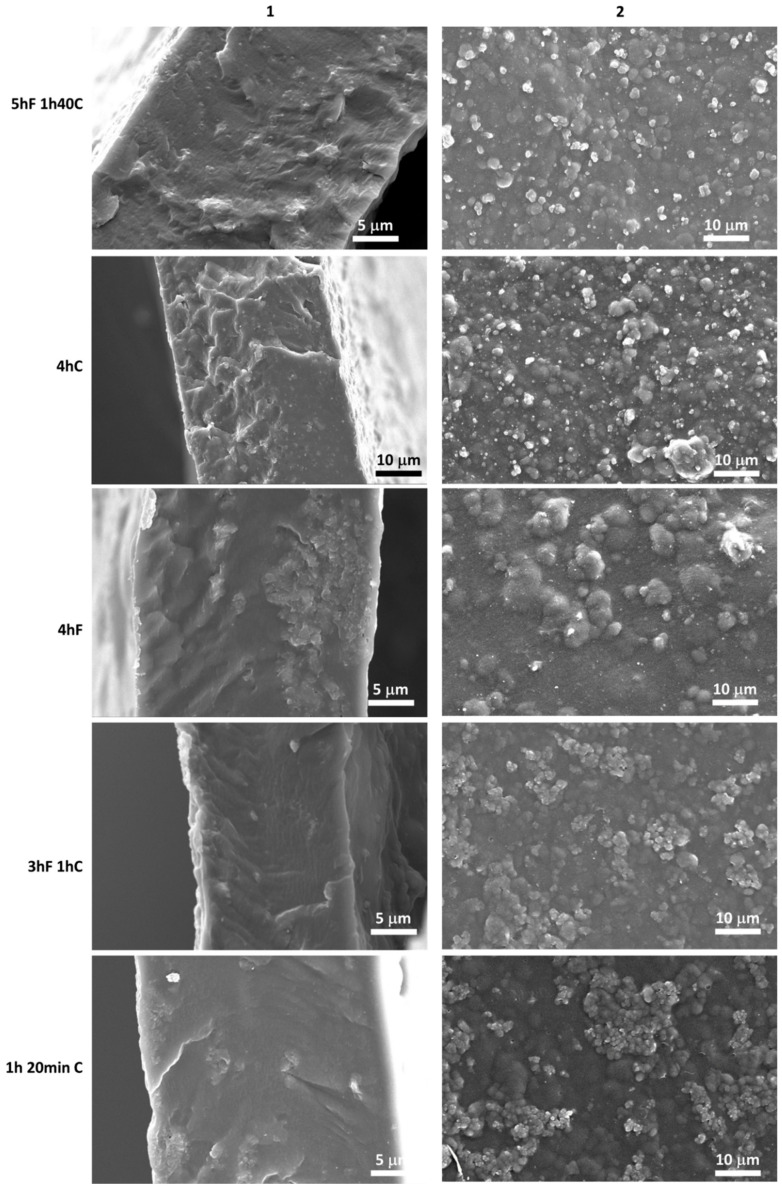
SEM images of cross-section (**1**) and surface (**2**) of MMMs at different ultrasound treatments.

SEM images of the prepared MMM membranes cast with different ultrasound treatments are presented in Figure 10.

The surface images in Figure 10 clearly illustrate the fillers’ presence on the surface of the membrane highlighting the dispersion of the filler over the membranes thickness. The aggregation is less visible across the cross section, except for 4hF. Despite the dispersion, varying levels of aggregation can be observed with the varying UT time. The membrane with the highest level of aggregation was for the membrane 4hF, as shown in Figure 10-4hF. The membranes 4hC and 5hF 1h40C displayed the lowest level of aggregation.

As shown in Figure S2, XRD patterns of the MMMs demonstrate that the crystallinity structure of ZIF-8 was well retained during the MMM preparation for all MMMs, even with the longer time of ultrasound.

#### 3.2.2. Separation Performances

**Figure 11:**
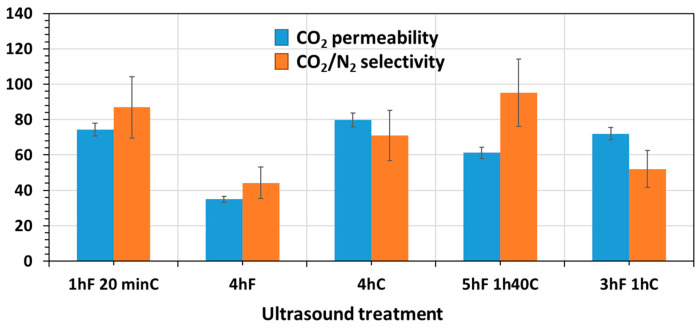
CO_2_ permeability (Blue) and CO_2_/N_2_ selectivity (Orange) of MMMs according to the ultrasound treatment. (The large error in the selectivity is due to the large uncertainty in N2 permeability).

Figure 11 shows the CO_2_ permeability and CO_2_/N_2_ selectivity of the MMMs cast with different ultrasound treatments. The data are summarized in Table S1. The 3hF 1hC corresponds to the MMM-25 °C.

The membrane with the longer time of UT (i.e., 5hF 1h40C) had the highest selectivity. Actually, particles can be reduced in size by the cavitation forces within ultrasound treatment. By keeping the volume fraction of filler the same, but decreasing the particle size, a greater number of smaller particles are created, providing a pathway through the membrane with a higher tortuosity. As a result, mass transfer resistance would increase leading to a reduced gas permeation and higher selectivity. Larger mass transfer resistance would occur for N_2_ due to its larger kinetic diameter.

The highest permeability was for the 4hC membrane at 80 Barrer. As shown in Figure 10, this membrane presented the best dispersion of the filler for all the membranes, which induces the maximum possible improvement. Using the Maxwell equation (See Supplementary Materials, SE1), the predicted CO_2_ permeability and CO_2_/N_2_ selectivity were calculated (Table 1).

As shown in Table 1, the predicted CO_2_ permeability is 81 Barrer, which is very close to the 4hC value (i.e., 80 Barrer), meaning that the interface between the filler and the polymer was without defect and optimal. The experimental selectivity was higher than the one predicted, probably due to the uncertainty of N_2_ permeability. In comparison with the normal procedure of fabricating the MMM, i.e., 3hF 1hC, the separation performance of 4hC was slightly improved from 72 Barrer to 80 Barrer and from 52 to 71 for the CO_2_ permeability and CO_2_/N_2_ selectivity.

The 4hF membrane had a significantly lower permeability and selectivity than the others MMMs of only 35 Barrer and 44, respectively. As suggested in Figure 10, the 4hF membrane showed high levels of aggregation, which might induce a possible pore blockage defect, and hence a decrease in permeability and selectivity [6]. With this procedure, there was no mixing between the filler and the polymer matrix, hence no possible dispersion of the filler within the polymer matrix.

These results shows that a long ultrasound time would lead to a lower permeability but a better selectivity due to the size reduction of the filler; the filler needed to be combined with ultrasound to be dispersed homogeneously and not create defects.

Compared to others MMMs (PEBAX based) (Table 2), the best MMMs prepared in this work, which correspond to the one casted at 50 °C and the 4hC, showed an intermediate CO_2_/N_2_ selectivity with a slightly lower CO_2_ permeability, which might be attributed to the differences in matrix polymer permeation and the operation conditions.

## 4. Conclusions

The purpose of this study was to investigate the effect of casting plate temperature and ultrasound treatment time and procedure to minimize the formation of defects, hence improving the MMMs performance and characteristics. The morphology and characteristics of the membranes were investigated to help understand defect formations and the best conditions to prevent them. For the effect of casting temperature, the PEBAX MH-1657/ZIF-8 MMMs were compared to neat PEBAX MH-1657 membranes. The SEM images of the neat PEBAX membranes highlighted a phase separation formation of the PEO and PA phases in the PEBAX. Spherulites consisting of randomly arranged lamellae PA crystals formed with increasing casting temperature. The impermeable spherulites caused blockages of the pores resulting in a lower permeability but maintaining selectivity in the membranes. The MMMs created successfully formed defect-free membranes at all of the casting temperatures. Addition of the filler succeeded in increasing the permeability and selectivity compared to the neat PEBAX membranes due to the increased FFV’s and ZIF-8′s selective adsorption of CO_2_. The casting temperature did not significantly impact the MMMs’ permeability and selectivity and achieved a high permeability and selectivity. The ultrasound treatment had a more significant effect on the formations of defects in the MMMs. While defects were observed in some membranes, defect-free membranes were also successfully produced. The best membranes produced were with the five-hour ultrasound treatment on the filler solution followed by one-hour ultrasound treatment on the combined solution and the four-hour ultrasound treatment on the combined solution. These presented the best filler dispersion and the smallest filler diameter, reducing aggregation, pore blockage and channeling.

## Figures and Tables

**Table 1:** Predicted CO_2_ permeability and CO_2_/N_2_ selectivity (ZIF-8 density = 0.95 g/cm^3^ [41]).

Materials	Permeability CO_2_ (Barrer)	Selectivity CO_2_/N_2_	Reference
PEBAX	49 (±3)	47 (±5)	Our work
ZIF-8	850	1.7	[43]
PEBAX_10%wtZIF-8	81	50	Calculated from Maxwell equation

**Table 2:** Separation performances of others MMMs based on PEBAX MH1657 matrix.

Materials	CO_2_ Permeability (Barrer)	CO_2_/N_2_ Selectivity	Measurement Conditions
PEBAX (This work)	49	47	1.2 bar and 25 °C
MMM_4hC (This work)	80	58	1.2 bar and 25 °C
MMM_50 °C (This work)	84	62	1.2 bar and 25 °C
PEBAX [41]	70	50	11 bar and 35 °C
PEBAX_ZIF8-5% [41]	130	46	11 bar and 35 °C
PEBAX_ZIF67-5% [41]	162	81	11 bar and 35 °C
PEBAX [34]	75	45	1 bar and 20 °C
PEBAX_ZIF8-10% [34]	120	52	1 bar and 20 °C
PEBAX [7]	70	34	3.75 bar and 25 °C
PEBAX_ZIF7-8% [7]	145	68	3.75 bar and 25 °C
PEBAX [16]	120	47	3 bar and 25 °C
PEBAX_ZIF8-10% [16]	175	41	3 bar and 25 °C
PEBAX [43]	45	60	1.2 bar and 25 °C
PEBAX_ZIF94-25% [43]	59	53	1.2 bar and 25 °C

## Data Availability

The data presented in this study are openly available in Edinburgh Data Share at https://doi.org/10.7488/ds/3468 (accessed on 6 February 2022).

## Supplementary Materials

The following supporting information can be downloaded at: https://www.mdpi.com/article/10.3390/membranes12060584/s1, Figure S1: High magnification SEM analysis of spherulite formation on neat PEBAX membrane cast at 35 °C; Figure S2: XRD patterns according casting plate temperatures (A and B) and ultrasound treatment (C); Figure S3: Thermograms of PEBAX according casting plate temperatures; Table S1: CO_2_ and N_2_ permeability, CO_2_ diffusivity and CO_2_ solubility coefficients for PEBAX and MMMs at different casting temperatures. SE1: Maxwell equation.
