# Supplementary material for: The Effect of Solution Casting Temperature and Ultrasound Treatment on PEBAX MH-1657/ZIF-8 Mixed Matrix Membranes Morphology and Performance
Source: Membranes (Basel). 2022 May 31;12(6):584. doi: 10.3390/membranes12060584 (PMC9228675; doi:10.3390/membranes12060584)
Supplement: Supplementary file 1 [file membranes-12-00584-s001.zip › membranes-1737595-supplementary.pdf]

membranes

Supplementary Information

The Effect Of Solution Casting Temperature And Ultrasound Treatment On PEBAX MH-1657/ZIF-8 Mixed Matrix Membranes Morphology And Performance

Elsa Lasseuguette [1], Louise Fielder-Dunton [1], Qian Jian[2] and Maria-Chiara Ferrari [1,*]

1 School of Engineering, University of Edinburgh, Robert Stevenson Road, Edinburgh EH9 3FB, UK
2 EaSTCHEM School of Chemistry, University of St Andrews, St Andrews KY16 9ST, UK
* Correspondence: m.ferrari@ed.ac.uk;

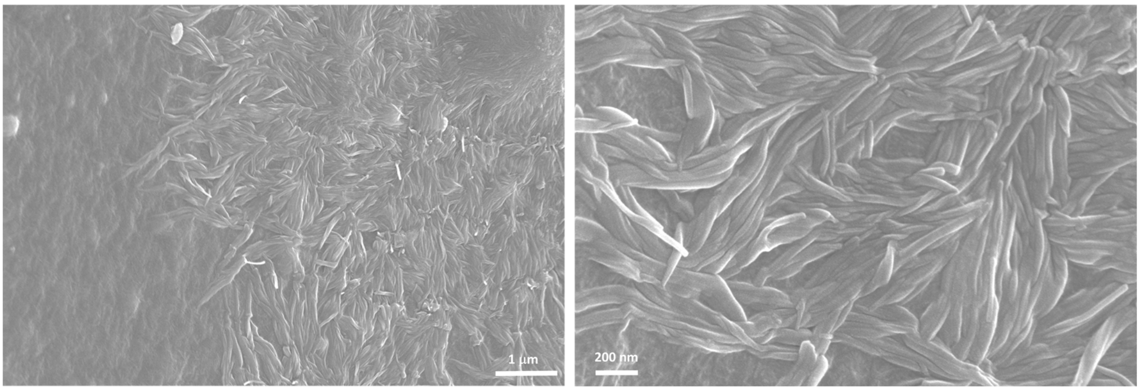

Figure S1: High magnification SEM analysis of spherulite formation on neat PEBAX membrane cast at 35 °C.

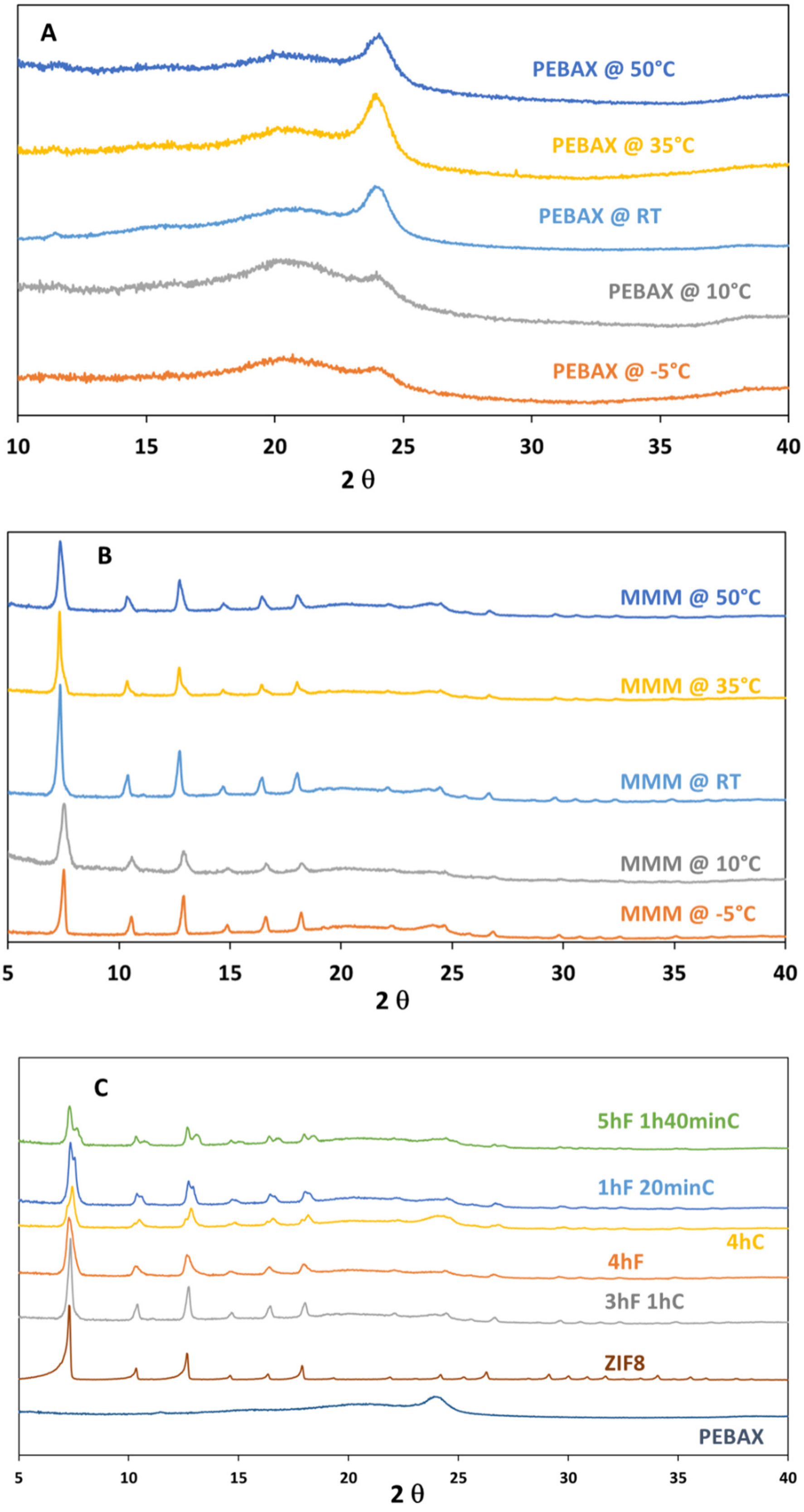

Figure S2: XRD patterns according casting plate temperatures (A and B) and ultrasound treatment (C).

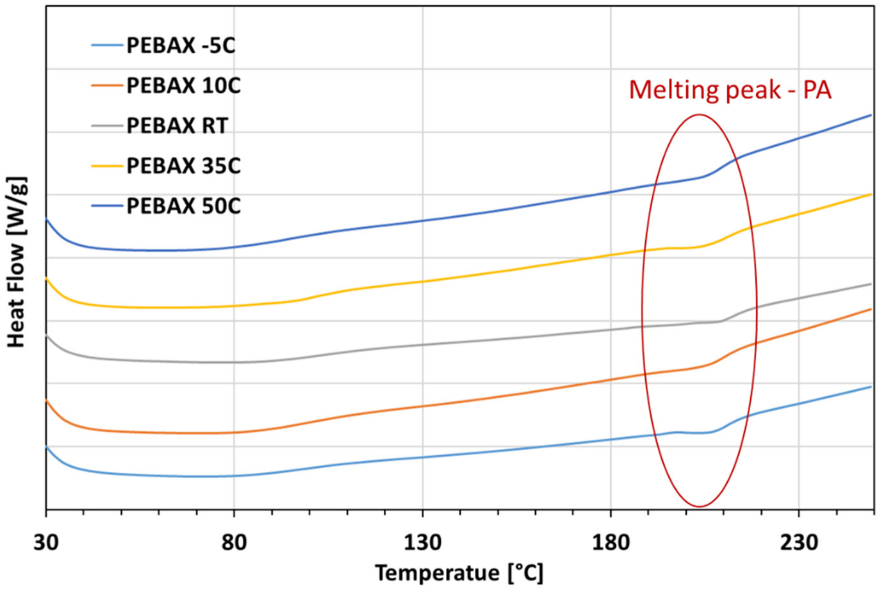

Figure S3: Thermograms of PEBAX according casting plate temperatures (*The curves have been shifted vertically for clarity*).

Table S1: CO_2 and N_2 permeability, CO_2 diffusivity and CO_2 solubility coefficients for PEBAX and MMMs at different casting temperatures. (*No data for N_2 diffusivity and solubility due to large uncertainty*).

Membrane	Permeability N_2 [Barrer]	Permeability CO_2 [Barrer]	Diffusivity CO_2 [10^{-7} cm²/s]	Solubility CO_2 [10^{-2} cm³(STP)/(cm³.cmHg)]
PEBAX – -5°C	6.2 (±1)	62 (±1)	2.3 (±0.1)	2.7 (±0.1)
PEBAX – 10°C	1.5 (±0.2)	73 (±2)	2.3 (±0.5)	3.2 (±1)
PEBAX – 25°C	1.1 (±0.5)	49 (±3)	2.6 (±0.3)	1.9 (±0.3)
PEBAX – 35°C	0.6 (±0.1)	37 (±1)	2.1 (±0.3)	1.7 (±0.2)
PEBAX – 50°C	0.4 (±0.2)	27 (±1)	2.8 (±0.9)	1 (±0.6)
MMM – -5°C	2.1 (±1)	66 (±2)	2.2 (±0.1)	3 (±0.1)
MMM – 10°C	1.1 (±0.1)	75 (±1)	2.1 (±0.3)	3.5 (±0.2)
MMM – 25°C	1.4 (±0.1)	72 (±1)	1.9 (±0.3)	3.8 (±0.7)
MMM – 35°C	1 (±0.3)	74 (±1)	2.2 (±0.1)	3.8 (±0.6)
MMM – 50°C	1.4 (±0.2)	84 (±1)	2.3 (±0.3)	3.6 (±0.4)
MMM – 1hF20minC	0.8 (±0.3)	74 (±1)	-	-
MMM – 4hF	0.8 (±0.4)	35 (±1)	-	-
MMM – 4hC	1.1 (±0.5)	80 (±1)	-	-
MMM – 5hF1h40C	0.7 (±0.3)	65 (±2)	-	-

Maxwell Equation:

$$P_{MMM} = P_P \left[\frac{P_{Filler} + 2P_P + 2\emptyset_{Filler}(P_{Filler} - P_P)}{P_{Filler} + 2P_P + \emptyset_{Filler}(P_{Filler} - P_P)} \right] \quad \text{(S1)}$$

where P_{MMM} is the gas permeability of the Mixed Matrix Membrane, P_P is the gas permeability of the polymer matrix (PEBAX), P_{Filler} is the gas permeability of the filler (ZIF-8), Φ_{Filler} is the volume fraction of the filler inside the MMM.
